# Sarcopenia in Interventional Radiology: An Opportunistic Imaging Biomarker for Patient Outcomes and Procedural Planning

**DOI:** 10.3390/muscles4040055

**Published:** 2025-11-13

**Authors:** Hyeon Yu

**Affiliations:** Division of Vascular and Interventional Radiology, Department of Radiology, University of North Carolina at Chapel Hill, 2018 Old Clinic, CB 7510, Chapel Hill, NC 27599, USA; hyeon_yu@med.unc.edu

**Keywords:** sarcopenia, interventional radiology, computed tomography, myosteatosis, prognosis, transarterial chemoembolization (TACE), transarterial radioembolization (TARE), transarterial embolization (TAE), transjugular intrahepatic portosystemic shunt (TIPS), radiofrequency ablation (RFA), peripheral arterial disease (PAD)

## Abstract

Sarcopenia, the loss of skeletal muscle mass and function, is a common and critical comorbidity in patients with conditions frequently managed by interventional radiologists, such as liver cirrhosis and hepatocellular carcinoma (HCC). Interventional radiologists are well positioned to incorporate opportunistic screening for this condition during routine preprocedural cross-sectional imaging. This review summarizes the current evidence on how sarcopenia influences patient outcomes and informs procedural planning across a spectrum of interventional radiology (IR) procedures. In transarterial embolizations for HCC, sarcopenia is a robust independent predictor of increased mortality, with meta-analyses suggesting it may also predict a lower tumor response rate. Even earlier stages of muscle loss (pre-sarcopenia) are associated with worse survival, and dynamic changes in muscle mass post-treatment can serve as a biomarker for tumor progression. For patients undergoing transjugular intrahepatic portosystemic shunt, pre-procedural sarcopenia and myosteatosis are strong, independent predictors of both mortality and the development of post-procedural hepatic encephalopathy, with the presence of both conferring the highest risk. In the context of pre-surgical portal vein embolization, sarcopenia is consistently associated with impaired volumetric liver growth, although this does not always translate to worse short-term surgical outcomes, as functional liver regeneration may be preserved. Following percutaneous liver tumor ablation, sarcopenia is a powerful predictor of overall mortality, while its role in predicting tumor recurrence remains an area of active investigation. Finally, in non-oncologic interventions for peripheral arterial disease, sarcopenia is highly prevalent and is associated with worse functional status, higher mortality, and a significantly increased risk of major amputation after endovascular therapy. In conclusion, sarcopenia is a powerful and readily available biomarker that provides crucial prognostic information—often independent of standard clinical scores—across a wide spectrum of IR procedures. The consistent evidence supports integrating sarcopenia evaluation into routine practice to enhance risk stratification, improve patient counseling, and guide multidisciplinary treatment planning.

## 1. Introduction

Sarcopenia is a progressive and generalized skeletal muscle disorder characterized by the accelerated loss of muscle mass and function [1,2]. It is a common and serious condition associated with adverse outcomes, including physical disability, poor quality of life, and increased mortality in both the general population and in patients with cancer [3,4,5,6,7,8]. Its negative impact is not limited to a single specialty; sarcopenia has been shown to be a consistent predictor of chronic disease progression, all-cause mortality, poorer functional outcomes, and postoperative complications across numerous medical and surgical fields [2,9,10]. Sarcopenia is particularly prevalent in patients with liver cirrhosis, a condition frequently encountered in interventional radiology (IR), with a reported incidence ranging from 30% to 70% in this population [11,12,13].

The clinical significance of sarcopenia is especially profound in patients with hepatocellular carcinoma (HCC), a primary malignancy often managed with image-guided locoregional therapies [14]. A large-scale meta-analysis of nearly 9000 patients demonstrated a pooled prevalence of sarcopenia in the HCC population of 42% [15]. Furthermore, its prognostic impact is substantial; another major meta-analysis found that in HCC patients undergoing curative-intent resection, the presence of sarcopenia more than doubled the risk of mortality (HR = 2.20) and significantly increased the risk of tumor recurrence (Recurrence-Free Survival HR = 1.50) [3].

Interventional radiologists are uniquely positioned to identify this critical comorbidity. Routine pre-procedural imaging, most commonly computed tomography (CT), is a cornerstone of modern interventional practice for treatment planning and patient assessment. This standard imaging provides an opportunity for opportunistic screening of sarcopenia by enabling objective, quantitative analysis of body composition without additional cost, radiation exposure, or deviation from the standard clinical workflow [16,17,18]. While existing meta-analyses have established that sarcopenia is a prognostic factor, this review aims to synthesize the evidence into a procedure-specific guide for practicing interventional radiologists. The practical implications of sarcopenia will be focused by exploring its impact across a range of common IR procedures, including transarterial endovascular therapies (TACE/TAE/TARE), transjugular intrahepatic portosystemic shunt (TIPS) creation, percutaneous radiofrequency ablation (RFA) of liver tumors, pre-surgical portal vein embolization (PVE), and endovascular interventions for peripheral arterial disease (PAD). Additionally, a practical summary of the various CT-based methods for assessing sarcopenia and myosteatosis will be provided to bridge the gap between radiological findings and actionable clinical decision-making.

## 2. Assessing Sarcopenia in the Interventional Radiology Setting

**Figure 1:**
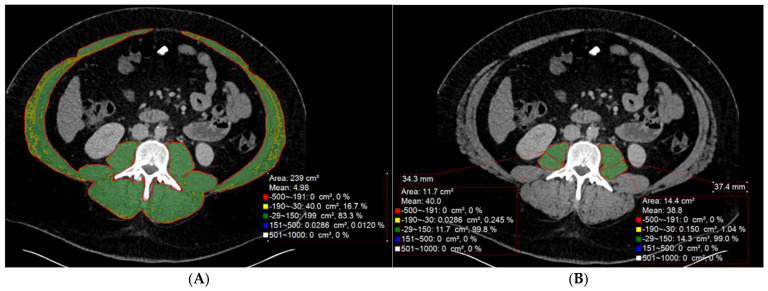
CT-Based Body Composition Assessment at the L3 Level. (**A**): The image demonstrates CT-based segmentation and assessment of the abdominal skeletal muscle area at the L3 level. (**B**): The image demonstrates CT-based segmentation and assessment of the psoas muscle areas, attenuations, and thicknesses.

The formal clinical diagnosis of sarcopenia, as defined by a consensus group, the European Working Group on Sarcopenia in Older People (EWGSOP), requires evidence of both low muscle mass and either low muscle strength or poor physical performance [2]. For the interventional radiologist, the primary contribution is the objective quantification of muscle mass and quality from imaging studies-a key component of full sarcopenia diagnosis. While other modalities, such as magnetic resonance imaging (MRI) and dual-energy X-ray absorptiometry (DEXA), can also be used to accurately assess muscle mass, CT is currently considered the gold standard for this purpose due to its availability, high resolution, and accuracy [2,12,19,20]. The most widely validated method for CT-based assessment is the measurement of the lumbar skeletal muscle index (L3-SMI) [3,21]. This technique involves segmenting the total cross-sectional area of all skeletal muscles at the mid-point of the third lumbar (L3) vertebra—including the psoas, erector spinae, quadratus lumborum, and abdominal wall muscles (transversus abdominis, internal and external obliques, and rectus abdominis) [19,22]—and normalizing this area (cm^2^) by the patient’s height in meters squared (m^2^). Figure 1A demonstrates an example of skeletal muscle segmentation and assessment at the L3 level. Muscle tissue is typically quantified using a standardized Hounsfield Unit (HU) threshold range of − 29 to +150 HU [19,22,23]. This segmentation is a recurring concern in clinical implementation, as manual tracing can be time-consuming and suffer from poor inter-observer reproducibility [24].

A further challenge is that, while the formal clinical definition of sarcopenia requires both low muscle mass and low muscle function, a significant portion of the published literature relies solely on imaging-based muscle mass measurements; one comprehensive literature review found this to be the case in nearly 70% of studies [2]. This is compounded by the lack of a universal, consensus-based diagnostic cutoff value for low muscle mass, as thresholds vary considerably by ethnicity, sex, and BMI [15,25]. For example, studies in Italian HCC cohorts have used cutoffs of ≤55 cm^2^/m^2^ for men and ≤39 cm^2^/m^2^ for women [23], whereas studies in Asian populations often use lower thresholds, such as the Japan Society of Hepatology guidelines of ≤42 cm^2^/m^2^ for men and ≤38 cm^2^/m^2^ for women [12,22,26,27]. Other definitions incorporate Body Mass Index (BMI) stratification, using different SMI thresholds for patients with a BMI above or below 25 kg/m^2^ [28,29]. It is also important to acknowledge that in patients with severe fluid retention, such as large-volume ascites or anasarca, CT-based attenuation values can be confounded, limiting assessment sensitivity. This heterogeneity underscores the importance of using population-appropriate, validated criteria when making a diagnosis. A summary of the various diagnostic criteria used in key studies is presented in Table 1.

**Table 1:** Various Sarcopenia Diagnostic Cutoffs in the Literature.

Reference	Population/Disease	Metric	Diagnostic Cutoff for Sarcopenia
Lanza et al. (2020) [23]	Italian/HCC (TAE)	L3-SMI	M: ≤55 cm^2^/m^2^ F: ≤39 cm^2^/m^2^
Rattanasupar et al. (2024) [22]	Thai/HCC (TACE)	L3-SMI	M: ≤42 cm^2^/m^2^ F: ≤38 cm^2^/m^2^ (JSH Guidelines)
Li et al. (2023) [12]	Chinese/Cirrhosis (TIPS)	L3-SMI	M: ≤42 cm^2^/m^2^ F: ≤38 cm^2^/m^2^ (JSH Guidelines)
Denbo et al. (2021) [28]	US/CLM (PVE)	L3-SMI	M: ≤53 cm^2^/m^2^ (if BMI > 25) OR < 43 cm^2^/m^2^ (if BMI < 25) F: ≤41 cm^2^/m^2^
Baby et al. (2025) [29]	Indian/Malignancy (PVE)	L3-SMI	M: <53 cm^2^/m^2^ (if BMI > 25) OR < 43 cm^2^/m^2^ (if BMI < 25) F: ≤41 cm^2^/m^2^
Stoffel et al. (2024) [30]	US/Cirrhosis (TIPS)	PMI	M: <4.36 cm^2^/m^2^ F: <3.23 cm^2^/m^2^ (Study-derived)
Kim et al. (2014) [11]	Korean/Cirrhosis (Ascites)	TPMT	M/F: ≤14 mm/m (at L4)
Yin et al. (2023) [31]	Chinese/Cirrhosis (TIPS)	TPMT	M: <10.7 mm/m F: <7.8 mm/m (at L3)
Yin et al. (2023) [31]	Chinese/Cirrhosis (TIPS)	PMA (Myosteatosis)	M/F: <41 HU (if BMI < 25) OR < 33 HU (if BMI ≥ 25)

L3 = 3rd Lumbar vertebral body; SMI = Skeletal Muscle Index; PMI = Psoas Muscle Index; TPMT = Transverse Psoas Muscle Thickness; PMA = Psoas Muscle Attenuation; TAE = Transarterial Embolization; TACE = Transarterial Chemoembolization; TIPS = Transjugular Intrahepatic Portosystemic Shunt; PVE = Portal Vein Embolization.

Given that L3-SMI segmentation can be time-consuming and may require dedicated software, several simplified methods that focus solely on the psoas muscle have been validated and are gaining popularity. One study evaluating conventional manual segmentation highlighted this issue, finding poor reproducibility between measurements and a low inter-rater agreement [24]. These include the Psoas Muscle Index (PMI), which normalizes the L3 psoas muscle area by height squared, and the Transverse Psoas Muscle Thickness (TPMT), a simple linear measurement [12,13,31]. Figure 1B demonstrates a simplified psoas segmentation and assessment. Multiple studies, particularly in the TIPS population, have shown that these psoas-only metrics are powerful and independent predictors of outcomes, such as mortality and hepatic encephalopathy (HE) [12,13,30,31]. However, it is important to recognize that psoas-only indices are surrogates, which may not always reflect whole-body muscle status, as atrophy can occur at different rates across muscle groups. For practical application, a key methodological study demonstrated that measuring psoas thickness at a fixed bony landmark, the L3 vertebra, is more reliable and consistent than using a mobile landmark, such as the umbilicus, especially in patients with ascites [12].

Beyond muscle quantity (mass), muscle quality—often assessed as myosteatosis (fatty infiltration)—is another critical component of muscle health that can be quantified on CT [13,31]. Myosteatosis is measured as the mean muscle attenuation (in HU) within a segmented muscle area, with lower HU values indicating greater fat infiltration [13,31]. Several studies in the TIPS population have shown that myosteatosis, measured, for example, by a low Psoas Muscle Attenuation (PMA), is an independent predictor of adverse outcomes, including HE and mortality [13,31]. Importantly, the negative effects of sarcopenia (low quantity) and myosteatosis (low quality) appear to be additive, with one study finding that patients with both conditions concurrently had the highest risk of post-TIPS complications and mortality [31]. The primary CT-based methods for assessing muscle quantity and quality are summarized in Table 2.

It is important to note that while CT provides the gold standard for muscle mass assessment, sarcopenia can also be effectively diagnosed using simple, non-imaging clinical tools. One recent prospective study in patients with chronic limb-threatening ischemia (CLTI) demonstrated that an algorithm combining the SARC-F questionnaire, handgrip strength, and BMI-adjusted calf circumference was a powerful, independent predictor of mortality and amputation [32]. This highlights that sarcopenia is a clinically relevant syndrome that can be identified through various means, reinforcing the importance of a multidisciplinary approach to patient assessment.

The practical implementation of these measurement techniques relies on various software solutions, many of which are documented in the literature. Commercially available and widely used software for semi-automated segmentation includes SliceOmatic (Version 5.0; Tomovision, Montreal, QC, Canada; https://www.tomovision.com, accessed on 11 October 2025) [12,19,22] and Osirix MD (Version 13.0; Pixmeo SARL, Geneva, Switzerland; https://www.osirix-viewer.com, accessed on 11 October 2025) [28]. For researchers and clinicians seeking a no-cost, open-source alternative, 3D Slicer (Version 5.6; https://www.slicer.org, accessed on 11 October 2025) has also been successfully used in multiple studies for this purpose [16,29,33]. Additionally, many modern Picture Archiving and Communication Systems (PACSs) or advanced visualization workstations may include proprietary tools for segmentation and analysis [23,24].

Finally, emerging concepts are expanding the role of imaging in sarcopenia assessment. A novel approach is the use of longitudinal or dynamic assessment, where the change (delta) in muscle mass on follow-up scans after an intervention is used as a biomarker [34]. One pilot study in TARE found that a decline in muscle mass at 3 months post-procedure was a reliable predictor of tumor progression, whereas a static baseline measurement was not [34]. To overcome the practical barriers of time-consuming manual measurements and their low reproducibility, Artificial Intelligence (AI)-based automated tools are being developed and validated [24,35]. These AI models have demonstrated high accuracy for diagnosing sarcopenia (sensitivity 82.3%, specificity 98.1%) [23]. Furthermore, the feasibility of implementing these tools in a real-world clinical setting has been demonstrated in a multicenter prospective study. In this study, a fully automated, PACS-integrated AI system performed screening in just a few seconds with high technical success and accuracy [20]. These technological advances promise to make body composition analysis a seamless, integrated, and routine part of the radiological workflow.

**Table 2:** Overview of CT-Based Methods for Assessing Muscle Quantity and Quality.

Metric	Description	Measures	Key Strengths/Weaknesses	Segmentation Software	References
L3 SMI	Cross-sectional area of all skeletal muscles at the L3 vertebral level, normalized by height squared (cm^2^/m^2^).	Muscle Quantity	Strength: Considered the imaging “gold standard” Weakness: Can be time-consuming; cutoffs vary by population.	SliceOmatic	Lanza et al. (2020) [23] Loosen et al. (2023) [33] Kong et al. (2024) [3] Salman et al. (2021) [19]
PMI/TPMT	Cross-sectional area or linear thickness of the psoas muscle(s), typically at L3, often normalized by height.	Muscle Quantity	Strength: Fast and simple “shortcut” with strong prognostic value Weakness: Less comprehensive than full SMI Best Practice: Measure at L3, not the umbilicus.	3D Slicer	Li et al. (2023) [12] Yin et al. (2023) [31] Stoffel et al. (2024) [30] Mukund et al. (2026) [13]
PMA	Mean radiodensity (in Hounsfield Units, HU) of the psoas muscle, reflecting fat content.	Muscle Quality (Myosteatosis)	Strength: Measures muscle quality, an independent and additive risk factor Weakness: Less established than quantity metrics.	3D Slicer ADW4.4	Mukund et al. (2026) [13] Yin et al. (2023) [31]
deltaPMI/deltaSMI	The dynamic change (increase or decrease) in a muscle metric (e.g., PMI) between baseline and a follow-up scan.	Muscle Trajectory	Strength: Serves as a dynamic biomarker of treatment response Weakness: Emerging concept; requires standardized follow-up.	NA	Trobiani et al. (2024) [34]
AI-Based Automation	Automated L3 selection and segmentation of muscle/fat	Quantity & Quality	Strength: Extremely fast, reproducible, and objective; enables large-scale screening Weakness: “Black box” nature; requires validation	iAID (In-house models)	Onishi et al. (2025) [24] Buttner et al. (2025) [35]

SMI = Skeletal Muscle Index; PMI = Psoas Muscle Index; TPMT = Transverse Psoas Muscle Thickness; PMA = Psoas Muscle Attenuation; deltaPMI = dynamic change in PMI; deltaSMI = dynamic change in SMI.

## 3. Impact of Sarcopenia on Outcomes in Specific Interventional Procedures

The negative impact of sarcopenia on prognosis has been established in the surgical literature for patients undergoing curative resection for HCC [3,36,37,38]. A growing body of evidence now demonstrates a similar, profound impact in patients treated with minimally invasive, image-guided therapies. This link is mechanistically critical, as sarcopenia is a surrogate for frailty, impaired immunity, and poor nutritional status, all of which compromise a patient’s ability to withstand the systemic inflammatory and metabolic stress of an interventional procedure and to subsequently heal and recover. This is particularly relevant given that the patient populations undergoing these procedures are often elderly, with many key studies reporting mean or median ages ranging from the mid-50s to early 70s [11,19,23,30,32]. The impact of sarcopenia on clinical outcomes varies by procedure and is summarized in Table 3. The following sections will review the role of sarcopenia in specific IR procedures.

### 3.1. Transarterial Embolizations (TAE/TACE/TARE)

The prognostic significance of sarcopenia in patients undergoing transarterial embolizations for liver malignancies is now well-established by a wealth of evidence, including several individual studies and a recent meta-analysis. The most consistent finding is the strong association between pre-procedural sarcopenia and long-term mortality. A 2024 meta-analysis of 12 studies involving over 2500 patients concluded that the presence of sarcopenia significantly increases the risk of death by approximately 46% (HR: 1.46) in patients treated with TAE/TACE [39]. Individual cohort studies support this finding; for example, a TACE study found that patients with low SMI had a median overall survival of only 404 days, compared with 1321 days for those without sarcopenia [33]. Similarly, in a cohort undergoing TAE, sarcopenia was a powerful, independent predictor of lower overall survival (HR = 2.22) [22]. The prognostic impact appears to begin even before overt sarcopenia develops. A recent study demonstrated that even pre-sarcopenia—defined as low muscle mass without functional decline—was an independent predictor of significantly shorter overall survival (18 vs. 30 months) after TACE [22].

While the link to mortality is clear, the impact of sarcopenia on tumor response to treatment is more complex. Several individual retrospective studies have reported that baseline sarcopenia or pre-sarcopenia does not predict the objective tumor response or the achievement of a complete response following TAE or TACE [22,23,33]. However, the larger, pooled meta-analysis by Long et al. did find a statistically significant, albeit modest, association between sarcopenia and a lower objective response rate [39]. This suggests that, while the effect may not be apparent in smaller individual cohorts, sarcopenia may indeed negatively influence the efficacy of embolization at a larger scale.

A novel perspective is offered by studies analyzing the dynamic changes in muscle mass after treatment. A pilot study on patients undergoing TARE with Holmium-166 found that the baseline sarcopenia status did not predict tumor response [34]. Instead, the worsening of sarcopenia at three months post-procedure was a reliable predictor of a poor loco-regional response and disease progression [34]. This suggests that the trajectory of a patient’s muscle mass following an intervention may serve as a dynamic biomarker of treatment efficacy and systemic response.

Importantly, despite its association with poor long-term survival, sarcopenia does not appear to increase the immediate risks of the procedure itself. One study explicitly found no significant difference in periprocedural complications, length of hospital stays, or 30-day readmission rates between sarcopenic and non-sarcopenic patients undergoing TAE [23]. Furthermore, while muscle quantity (sarcopenia) is a robust predictor, muscle quality (myosteatosis) appears less significant in this context. Both an individual study and the large meta-analysis found that myosteatosis was not independently associated with overall survival after TACE [33,39].

### 3.2. Percutaneous Tumor Ablation

Percutaneous ablation, most commonly RFA, is a curative-intent therapy for early-stage HCC [40]. While the procedure has high rates of technical success and local tumor control, the patient’s underlying health status, particularly their muscle mass, plays a significant role in long-term outcomes. The available evidence strongly indicates that sarcopenia is a powerful predictor of mortality in this patient population. A prospective study by Salman et al. found that sarcopenia was the strongest independent predictor of two-year mortality after RFA for HCC, increasing the risk of death by nearly eightfold (HR = 7.6) [19]. The survival difference was stark: the two-year overall survival rate for sarcopenic patients was only 36.6%, compared to 85.5% for their non-sarcopenic counterparts [19]. This finding is reinforced by a large meta-analysis on curative treatments for HCC (including RFA), which concluded that sarcopenia more than doubled the risk of long-term mortality (HR = 2.20) [3].

In contrast, the relationship between sarcopenia and the risk of tumor recurrence after RFA is less clear, with conflicting reports in the literature. The prospective study by Salman et al. found no significant association between sarcopenia and recurrence-free survival, suggesting that while frail patients died sooner, it was not necessarily due to a higher rate of tumor recurrence [19]. Conversely, a retrospective study by Jaruvongvanich et al. found a potential association, reporting that sarcopenic patients had a significantly shorter median time to recurrence (17.6 vs. 36.7 months) [41]. Although sarcopenia was not an independent predictor in their final multivariate analysis, the result was borderline (*p* = 0.052), leading the authors to describe it as a potential prognostic factor [41]. Lending further support to this potential link, the large meta-analysis by Kong et al., which included RFA patients, did find a significant association between sarcopenia and worse recurrence-free survival (HR = 1.50) [3].

Taken together, these findings suggest that sarcopenia unequivocally predicts poor overall survival in patients undergoing RFA, reflecting the increased risk of death from the complications of underlying liver disease and frailty, even after a successful local tumor treatment. The potential link to higher tumor recurrence, supported by a large meta-analysis but not consistently found in smaller individual studies, suggests a more complex relationship that may involve host immune surveillance or tumor biology and warrants further investigation. For practicing interventional radiologists, these data underscore the importance of assessing sarcopenia on pre-ablation imaging. This assessment provides critical prognostic information for patient counseling, helping manage expectations for long-term survival, even when a complete and successful ablation is anticipated.

### 3.3. Portal Vein Embolization (PVE)

The PVE is a critical preoperative procedure performed by interventional radiologists to induce hypertrophy of the future liver remnant (FLR), thereby enabling safe and extensive hepatectomy for liver tumors [42,43]. The patient’s underlying nutritional and metabolic state, as reflected by sarcopenia, has been shown to significantly influence the hepatic regenerative response to this procedure. The most consistent finding across multiple studies is a clear association between sarcopenia and an impaired volumetric growth of the FLR after PVE [16,28,29,44]. Patients with lower muscle mass consistently demonstrate lower hypertrophy and slower kinetic growth rate (KGR) [16,28,29,44]. The role of visceral fat in this process is also an area of interest, though the evidence is less consistent; one study found that a low visceral adipose index (VAI) was strongly associated with poor liver hypertrophy [28], while another found only a weak, non-significant correlation [29].

While the general principle that lower muscle mass predicts poorer volumetric growth is well established, the optimal imaging metric for this prediction remains under investigation. Most studies have successfully used the standard L3-SMI to demonstrate this association [28,29,44]. However, one study found that a volumetric measurement of the psoas muscle (psoas muscle volume, PMV) was a stronger predictor of KGR than L3-SMI, which was not significant in their analysis [16]. This suggests that different body composition metrics may have varying predictive power depending on the specific clinical question and patient population.

Perhaps the most important and clinically relevant finding in this area is the potential dissociation between volumetric and functional liver regeneration. A recent study utilizing hepatobiliary scintigraphy (HBS) to directly measure liver function made a crucial observation: while sarcopenic patients had the expected impairment in volumetric growth (KGR), their functional growth rate (FGR) was preserved [44]. This suggests that in sarcopenic patients, the liver remnant may be increasing its functional capacity per unit of volume, compensating for the lack of anatomical hypertrophy.

This dissociation between volume and function may explain the seemingly paradoxical finding that impaired volumetric growth in sarcopenic patients does not necessarily lead to worse short-term surgical outcomes. In line with the preserved functional growth, two independent studies found no significant difference in the rates of successful surgical resection, postoperative complications, or 90-day mortality between sarcopenic and non-sarcopenic patients who had undergone PVE [29,44]. This has led to the hypothesis that the PVE procedure itself may act as a protective factor, successfully preparing even frail, sarcopenic patients for major hepatectomy [29]. For the interventional radiologist, this implies that while a sarcopenic patient is likely to have a slower volumetric response, this finding alone should not preclude them from surgery, and a direct assessment of liver function may be more informative than volumetry for determining surgical readiness.

### 3.4. Transjugular Intrahepatic Portosystemic Shunt (TIPS)

The TIPS is a procedure for managing severe complications of portal hypertension, such as refractory ascites, hydrothorax, and variceal hemorrhage [45,46]. Given the inherent frailty of this patient population, risk stratification is paramount, and sarcopenia has emerged as a key independent predictor of post-procedural outcomes. The most robustly demonstrated impact of sarcopenia is on post-TIPS mortality. Recent multiple studies have independently confirmed that pre-procedural sarcopenia is a powerful predictor of short-term and one-year mortality, even after accounting for standard measures of liver dysfunction [12,30,31,47]. One study found that sarcopenic patients had a 2.4-fold higher risk of death within the first year after TIPS placement [30]. This prognostic value holds true whether sarcopenia is assessed using the comprehensive L3-SMI or simpler, psoas-only metrics [12,30], and it aligns with earlier findings in the broader population of cirrhotic patients with ascites [11].

Beyond mortality, sarcopenia is also a strong predictor of HE, one of the most common and debilitating complications of the TIPS procedure. This relationship is biologically plausible, as skeletal muscle plays a key role in extrahepatic ammonia metabolism, and sarcopenia has been linked to HE in the broader cirrhotic population [2]. Several studies have now established that pre-TIPS sarcopenia is an independent risk factor for the development of new or worsening overt HE [13,31,35]. Reduced muscle mass impairs the body’s ability to clear ammonia—precisely the toxin that is diverted into systemic circulation by the TIPS—thereby exacerbating the risk of HE.

Further research has refined this association by differentiating between muscle quantity (mass) and muscle quality (myosteatosis). Both sarcopenia and myosteatosis, measured on CT as low psoas muscle mass and low PMA, respectively, have been identified as independent predictors of both post-TIPS HE and overall mortality [13,31]. Crucially, their negative effects appear to be additive. One study demonstrated a clear risk gradient, where patients with both sarcopenia and myosteatosis had the highest incidence of HE and the worst survival, patients with only one condition had intermediate risk, and patients with neither had the best outcomes [31].

The prognostic information provided by sarcopenia is not redundant with existing clinical scores but provides a distinct and complementary axis of risk. It has been shown that sarcopenia’s predictive power for mortality is independent of and adds to that of the Model for End-Stage Liver Disease (MELD) score [30]. This has led to the development of combined prognostic models; a recent study created a MELD-Na/sarcopenia model that demonstrated significantly superior accuracy for predicting one-year mortality compared to using either MELD-Na or sarcopenia in isolation [47]. This study highlighted that sarcopenia dramatically worsens prognosis at every level of MELD-Na, effectively moving patients in an intermediate MELD-Na risk category into a high-risk category for mortality [47].

Perhaps the most exciting and novel concept emerging from recent literature is the potential for the TIPS procedure to actively reverse sarcopenia. One study demonstrated a significant improvement in both muscle quantity (SMI and PMI) and muscle quality (PMA) on follow-up imaging after TIPS placement [13]. This suggests that by reducing portal hypertension and improving systemic hemodynamics and nutrient absorption, TIPS may be a therapeutic intervention for sarcopenia itself. However, this finding is not yet universally established. A separate study using an AI-based analysis tool found no clear trend in post-TIPS muscle changes, though the authors noted that heterogeneity in follow-up imaging times may have obscured a potential effect [35]. This remains a promising but unresolved area requiring further prospective investigation. From a practical standpoint, the literature supports the use of simplified, psoas-only measurements as reliable and efficient prognostic tools in this population [12,30,31], with a key recommendation to perform measurements at a fixed bony landmark, such as the L3 vertebra, for maximal consistency [12].

### 3.5. Peripheral Artery Disease (PAD) Interventions

Beyond the realm of hepatobiliary and oncologic interventions, sarcopenia has also been established as a critical prognostic factor in patients undergoing endovascular therapy (EVT) for PAD. Sarcopenia is a highly prevalent comorbidity in this population; a systematic review found a pooled prevalence of approximately 35%, a prospective study of patients with CLTI found a prevalence of 45.3%, and another study in men with PAD found the prevalence to be nearly 10 times greater than in age- and BMI-matched controls without the disease [25,32,48]. The coexistence of these two conditions creates a synergistic negative impact on a patient’s functional status. For instance, patients with both PAD and sarcopenia demonstrate significantly worse mobility, including a shorter 6-min walk distance and a longer recovery time from claudication pain after exercise, when compared to PAD patients without sarcopenia [48].

For the interventional proceduralist, the most critical finding is the profound impact of sarcopenia on post-procedural outcomes. A recent study on patients undergoing peripheral EVT for PAD found dramatically worse outcomes in the sarcopenic group. Sarcopenic patients had significantly higher rates of both mortality (65.7% vs. 20%) and major amputation (57.1% vs. 15.4%) during follow-up [49]. This finding is supported by prospective data showing that sarcopenia, even when diagnosed using simple clinical tools, such as calf circumference and handgrip strength, is an independent predictor for a composite outcome of 6-month mortality and/or major amputation (HR 1.95) in patients with CLTI [32]. This strong association with mortality is supported by several reviews, which conclude that lower skeletal muscle mass is a consistent predictor of a poor prognosis in the broader PAD population [25,50].

The pathophysiological link between PAD and sarcopenia is a vicious cycle. The chronic muscle ischemia inherent to PAD creates a local environment of inflammation, oxidative stress, and mitochondrial dysfunction, all of which are powerful drivers of muscle wasting and the development of sarcopenia [25]. This systemic frailty, in turn, contributes to the poor wound healing and reduced resilience that lead to higher rates of amputation and mortality following an intervention. Therefore, identifying sarcopenia on a pre-procedural CT angiogram is a crucial step in risk stratification. This simple, opportunistic assessment can flag patients at an exceptionally high risk for limb loss and death following limb salvage procedures, providing vital information for patient counseling.

### 3.6. Methodological Considerations and Limitations of Current Evidence

While the evidence presented in this review is compelling, it is essential to critically evaluate the methodological limitations. Most of the studies cited are single-center, retrospective observational analyses. This study design, while excellent for hypothesis generation and identifying associations, carries an inherent risk of selection bias and confounding variables. Furthermore, it limits the ability to establish definitive causation. For example, it is difficult to determine conclusively if sarcopenia is a direct cause of poor outcomes (e.g., impaired liver regeneration) or if it is a powerful surrogate marker for other unmeasured factors, such as advanced malnutrition, systemic inflammation, and biologic frailty, that are the true drivers of mortality and complications.

The level of evidence for most findings is, therefore, predominantly based on cohort and case–control studies. While a formal GRADE (Grading of Recommendations, Assessment, Development and Evaluations) assessment is beyond the scope of this narrative review, the field currently lacks high-level evidence from multi-center prospective studies and, most importantly, randomized controlled trials (RCTs) [51]. This gap is particularly evident in the field of IR, where the concept of “prehabilitation” (proactive nutritional and exercise therapy) is based on logical inference rather than high-quality trial data. Therefore, the conclusions and recommendations presented in this review should be interpreted with caution, and they serve as a strong call to action for future, more rigorous prospective investigation.

**Table 3:** Summary of Sarcopenia’s Impact on Outcomes in Key Interventional Radiology Procedures.

IR Procedure	Primary Outcomes Assessed	Key Findings	Key References
TAE, TACE, TARE	OS, Tumor Response, Complications	Predicts worse OS Not associated with tumor response in individual studies Worsening sarcopenia post-TARE predicts tumor progression Not associated with increased procedural complications	Lanza et al. (2020) [23] Loosen et al. (2023) [33] Rattanasupar et al. (2024) [22] Long et al. (2024) [39] Trobiani et al. (2024) [34]
TIPS	OS, HE, Post-procedural muscle changes	Predicts worse OS and development of HE Risk is highest when combined with myosteatosis Prognostic value is additive to MELD score Conflicting evidence on reversal of sarcopenia post-procedure.	Li et al. (2023) [12] Mukund et al. (2026) [13] Büttner et al. (2025) [35] Stoffel et al. (2024) [30] Delgado et al. (2024) [47] Yin et al. (2023) [31]
PVE	KGR, FGR, Surgical Outcomes	Predicts impaired KGR of the liver remnant FGR may be preserved. Does not predict worse short-term surgical outcomes	Schulze-Hagen et al. (2020) [16] Denbo et al. (2021) [28] Arntz et al. (2024) [44] Baby et al. (2025) [29]
RFA	OS, RFS	Strongly predicts worse OS Conflicting evidence on predicting tumor recurrence	Salman et al. (2021) [19] Jaruvongvanich et al. (2023) [41] Kong et al. (2024) [3]
PAD Interventions	Mortality, Major Amputation, Functional Status	Predicts higher mortality and major amputation rates after endovascular therapy Associated with worse functional status	Addison et al. (2018) [48] Dagli et al. (2025) [49] Pizzimenti et al. (2020) [25] Ferreira et al. (2021) [50]

TAE = Transarterial Bland Embolization; TACE = Transarterial Chemoembolization; TARE = Transarterial Radioembolization; TIPS = Transjugular Intrahepatic Portosystemic Shunt; PVE = Portal Vein Embolization; RFA = Radiofrequency Ablation; PAD = Peripheral Artery Disease; OS = Overall Survival; HE = Hepatic Encephalopathy; KGR = Volumetric Liver Growth; FGR = Functional Liver Growth; RFS = Recurrence Free Survival; MELD = Model for End-Stage Liver Disease.

## 4. Integrating Sarcopenia into Clinical Practice and Procedural Planning

The growing body of evidence demonstrates that the profound prognostic impact of sarcopenia necessitates its integration into the routine clinical practice of IR. The identification of sarcopenia is not merely an academic observation but a clinically actionable finding that can enhance risk stratification, improve patient counseling, and guide multidisciplinary management.

### 4.1. The Feasibility of Opportunistic Screening

Routinely performed pre-procedural CT imaging provides interventional radiologists with a unique opportunity to incorporate opportunistic sarcopenia screening into clinical practice [16]. This assessment can be integrated into the standard interpretation of scans performed for procedural planning, adding significant value without additional cost, imaging time, or radiation exposure to the patient. While the gold-standard L3-SMI can be laborious, validated “shortcut” methods, such as measuring psoas muscle thickness or index at the L3 level, provide powerful prognostic information and are highly feasible within a busy clinical workflow [12,30,31]. The primary barriers to widespread adoption—the time-consuming nature and poor reproducibility of manual segmentation—are now being overcome by emerging technologies [24]. The development of AI-based automated tools promises to make screening nearly effortless. Indeed, a recent prospective study of a fully automated, PACS-integrated AI system demonstrated 100% technical success in performing opportunistic screening, with an average processing time of 4.12 s per scan and an accuracy of 97.4% compared to human experts [20]. Such tools can make body composition analysis a seamless and objective part of the daily workflow.

### 4.2. A Powerful Tool for Prognostication and Risk Stratification

Across a wide range of procedures—including TAE/TACE/TARE, RFA, PVE, TIPS, and peripheral EVT—sarcopenia is a robust and independent predictor of overall mortality [3,19,30,33,39,49,52]. A critical aspect of its value is that this prognostic information is often complementary to and independent of standard clinical and laboratory scores. For example, in the TIPS population, sarcopenia’s predictive power for mortality remains significant even after adjusting for the MELD score [30]. This indicates that sarcopenia captures a distinct axis of risk related to frailty and physiologic reserve that is not reflected in measures of organ function alone. This has led to the development of superior combined prognostic models.

### 4.3. Enhancing Patient Counseling and Shared Decision-Making

The integration of objective, quantitative data on muscle mass into the pre-procedural workup can profoundly enhance patient counseling and shared decision-making. Moving beyond a subjective clinical impression of frailty, the interventional radiologist can use this imaging biomarker to have a more transparent and evidence-based discussion about personalized risk. For instance, counseling a patient with PAD is fundamentally changed when they can be informed that their sarcopenic status is associated with a post-intervention major amputation rate of over 50% and a mortality rate of over 65% [49]. Similarly, a candidate for TIPS can better understand their personal risk profile when informed that the presence of both sarcopenia and myosteatosis confers the highest risk for developing post-procedural HE [31]. This level of detail allows patients and their families to make more informed decisions that align with their goals of care.

### 4.4. Guiding Multidisciplinary Treatment Planning

Identifying sarcopenia can directly influence multidisciplinary treatment strategies. In the setting of PVE, a sarcopenic patient with an expectedly poor volumetric growth rate (KGR) may not be a poor surgical candidate. Knowing that their functional growth (FGR) is likely preserved can lead the team to incorporate direct functional imaging, such as HBS, to more accurately determine surgical readiness, rather than relying on volume alone [44]. For TIPS, the finding that pre-procedural sarcopenia and myosteatosis are high-risk factors for post-procedural HE might prompt the team to use a smaller-diameter stent to mitigate this risk [13,31]. In TARE, the finding that a worsening of sarcopenia on a 3-month follow-up scan predicts tumor progression can serve as an early, objective signal to the oncology team that the local therapy may be failing and that a switch to systemic therapy should be considered [34].

Beyond high-level planning, severe sarcopenia has immediate, practical implications for the procedure itself. Patients with advanced sarcopenia and cachexia often have minimal subcutaneous and intramuscular tissue, which can obscure normal palpable and sonographic landmarks for percutaneous access. Furthermore, these frail patients may have greater difficulty remaining immobile for the duration of a procedure, such as a lengthy ablation or embolization. Identifying these high-risk patients in advance allows the interventional team to plan accordingly, for example, by allocating additional procedure time or arranging for enhanced anesthetic support to ensure patient stability and procedural success.

### 4.5. Initiating Proactive Intervention: Prehabilitation

**Figure 2:**
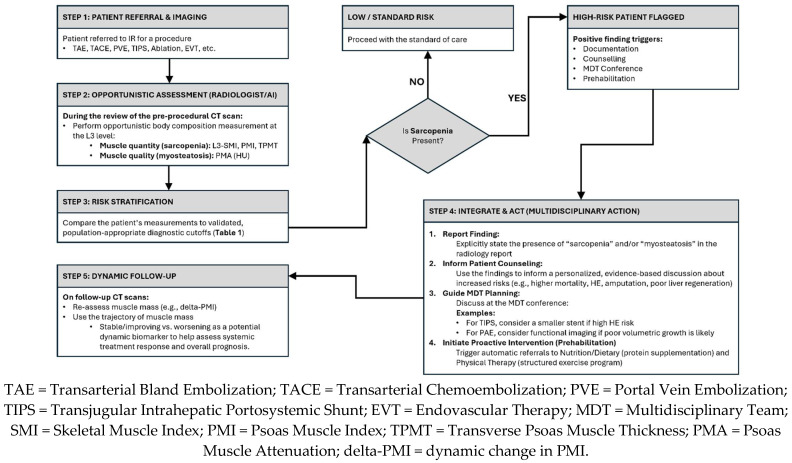
Proposed Clinical Algorithm for Opportunistic Sarcopenia Screening in Interventional Radiology.

Ultimately, the goal of identifying sarcopenia should not be simply to label a patient as high-risk, but to initiate action. Sarcopenia is a modifiable risk factor. The identification of low muscle mass—even at the early stage of pre-sarcopenia [22]—on a pre-procedural scan should trigger a multidisciplinary prehabilitation consult. This proactive approach involves early referrals to nutrition services for counseling on protein intake and to physical therapy for structured, personalized exercise programs designed to build physiologic reserve before the stress of an intervention [28]. The emerging, though debated, evidence that some IR procedures may improve muscle mass further highlights the dynamic interplay between the intervention and the patient’s systemic health, reinforcing the importance of a holistic, patient-centered approach to care [13,35]. Figure 2 demonstrates a proposed clinical algorithm for opportunistic sarcopenia screening in IR.

## 5. Future Directions

While the evidence establishing sarcopenia as a key prognostic biomarker in IR is compelling, the field is rapidly evolving from observation to intervention. The current body of literature, though robust, is mainly retrospective and has illuminated several key areas of debate and opportunity that warrant further investigation.

A foundational need for the field is the standardization of diagnostic criteria. This review highlights significant heterogeneity in CT-based cutoff values used to define sarcopenia, with thresholds varying by ethnicity, sex, and BMI stratification [12,23,28,29]. Furthermore, debate exists regarding the optimal metric, whether it be the comprehensive L3-SMI or a simplified psoas-only measurement [12,16]. Future research, ideally through large, multi-ethnic consortia, should aim to establish consensus-based, population-specific, and, perhaps, procedure-specific diagnostic thresholds for both sarcopenia and myosteatosis. Building on this, future investigations should also aim to improve current scoring systems, perhaps by integrating these validated imaging biomarkers with functional data and serum markers to create more comprehensive, clinically relevant prognostic models. This will be critical for improving the comparability of data across studies and for the confident implementation of these metrics in clinical guidelines.

The most important next step for the field is to move beyond prognostic observation and toward intervention. Most of the evidence to date is from retrospective studies, and there is a clear need for large-scale, multicenter prospective studies to confirm these findings and minimize selection bias [29,35]. More importantly, the central question of whether sarcopenia is a modifiable risk factor in the IR population must be addressed through randomized controlled trials (RCTs). These trials are urgently needed to test the efficacy of prehabilitation strategies [22]. Such studies should investigate whether targeted interventions—including nutritional supplementation and structured physical therapy—initiated upon the radiological identification of sarcopenia can improve muscle mass and, critically, whether this translates into improved clinical outcomes, such as reduced complication rates and better long-term survival after IR procedures. A key question for these trials will be to determine whether a short-term delay (e.g., 2–4 weeks) of non-emergent procedures, such as elective TACE or PVE, to allow for a prehabilitation program, results in superior long-term outcomes, thereby providing the evidence needed to justify a change in the clinical care pathway.

Several specific clinical questions and controversies arising from this review also warrant further investigation. The exciting yet conflicting findings on sarcopenia reversal after TIPS represent a key area for future research [13,35]. Prospective studies with standardized imaging follow-up protocols are essential to definitively determine if, and to what extent, the TIPS procedure itself can improve a patient’s systemic muscle health. Similarly, the dissociation between impaired volumetric growth and preserved functional growth in sarcopenic patients after PVE warrants further exploration [44]. Studies combining CT volumetry with functional liver imaging could clarify whether functional assessment should become the primary determinant of surgical readiness in this population. Finally, the potential link between sarcopenia and tumor biology, suggested by findings of lower tumor response or higher recurrence in some studies of TACE and RFA, is another avenue for translational research [3,39,41].

Finally, future research should aim to broaden the scope of investigation beyond the current focus on hepatobiliary disease. There is a notable gap in the literature regarding the impact of sarcopenia on outcomes in other major domains of IR, such as non-hepatic oncologic interventions (e.g., renal and lung ablation) and emergency procedures (e.g., trauma and GI bleed embolization), where patient frailty is equally critical. To facilitate future work, the continued development and clinical validation of AI-based tools for automated body composition analysis will be crucial. By making sarcopenia assessment a rapid, seamless, and integrated part of the routine radiological workflow, this technology will be the engine that drives the large-scale research needed to answer these important questions [35].

## 6. Conclusions

Sarcopenia is a highly prevalent, prognostically significant, and frequently overlooked comorbidity in patients undergoing care in IR. The evidence provided in this review demonstrates that imaging-diagnosed sarcopenia profoundly impacts outcomes across a wide spectrum of oncologic and vascular procedures. As an opportunistic biomarker readily available on routine pre-procedural imaging, its assessment represents a high-value addition to the standard radiological workup. The impact of sarcopenia is consistent and clinically significant. It is a robust predictor of overall mortality after transarterial therapies, RFA, and TIPS, often providing prognostic information that is independent of and additive to standard scores, such as MELD. It is also a key risk factor for major complications, including post-TIPS HE and post-EVT major amputation. Furthermore, it serves as an important predictor of the body’s regenerative capacity, as seen in its impairment of volumetric liver growth after PVE. The findings of this review constitute a clear call to action for the IR community. It is time to move beyond a purely lesion-focused interpretation of scans and to adopt a more holistic, patient-centered approach that incorporates an assessment of overall health. The routine integration of body composition analysis into the daily radiological workflow is a practical and powerful step toward this goal. By identifying and reporting sarcopenia, interventional radiologists can significantly improve risk stratification, enhance shared decision-making through more personalized patient counseling, and, most importantly, trigger proactive multidisciplinary interventions such as prehabilitation. Ultimately, recognizing and acting upon this critical biomarker is essential to improving the resilience of patients and their outcomes following IR procedures. The inclusion of standardized sarcopenia reporting should be advocated as a routine component of pre-procedural imaging checklists in IR.

## Data Availability

No new data were created or analyzed in this study.
